# Validation of Addenbrooke’s cognitive examination III for detecting mild cognitive impairment and dementia in Japan

**DOI:** 10.1186/s12877-019-1120-4

**Published:** 2019-04-29

**Authors:** Shintaro Takenoshita, Seishi Terada, Hidenori Yoshida, Megumi Yamaguchi, Mayumi Yabe, Nao Imai, Makiko Horiuchi, Tomoko Miki, Osamu Yokota, Norihito Yamada

**Affiliations:** Department of Neuropsychiatry, Okayama University Graduate School of Medicine, Dentistry and Pharmaceutical Sciences, 2-5-1 Shikata-cho, Kita-ku, Okayama, 700-8558 Japan

**Keywords:** Addenbrooke’s cognitive examination, Cognitive screening, Diagnosis dementia, Diagnosis mild cognitive impairment, Mild cognitive impairment

## Abstract

**Background:**

Early detection of mild cognitive impairment (MCI) and dementia is very important to begin appropriate treatment promptly and to prevent disease exacerbation. We investigated the screening accuracy of the Japanese version of Addenbrooke’s Cognitive Examination III (ACE-III) to diagnose MCI and dementia.

**Methods:**

The original ACE-III was translated and adapted to Japanese. It was then administered to a Japanese population. The Hasegawa Dementia Scale-revised (HDS-R) and Mini-mental State Examination (MMSE) were also applied to evaluate cognitive dysfunction. In total, 389 subjects (dementia = 178, MCI = 137, controls = 73) took part in our study.

**Results:**

The optimal ACE-III cut-off scores to detect MCI and dementia were 88/89 (sensitivity 0.77, specificity 0.92) and 75/76 (sensitivity 0.82, specificity 0.90), respectively. ACE-III was superior to HDS-R and MMSE in the detection of MCI or dementia. The internal consistency, test-retest reliability, and inter-rater reliability of ACE-III were excellent.

**Conclusions:**

ACE-III is a useful cognitive test to detect MCI and dementia. ACE-III may be widely useful in clinical practice.

## Background

Early detection of cognitive deterioration in the prodromal stage of dementing diseases is arguably important in order to initiate curable treatments in the future. Mild cognitive impairment (MCI) converts to dementia at a rate of ~ 10% per year [1], but its clinical diagnosis is a challenge due to the variety and often dynamic nature of symptoms [2]. Nonetheless, a reliable and valid test to detect MCI in clinical settings has not been developed [3].

ACE and its revised version (ACE-R) were created as concise tests for detecting mild cognitive dysfunction [4, 5]. ACE-R has been translated for use in a number of non-English-speaking countries worldwide and widely adopted in clinical and research settings [6]. A study at our facility verified the Japanese version [7]. Despite its widespread use, ACE-R is relatively weak in several domains, such as repetition, comprehension, and visuospatial items [2, 6]. Healthy older adults often fail the repetition item on ACE-R due to poor hearing or a short attention span [8]. Comprehension items on ACE-R exhibit poor sensitivity to cognitive impairment because individuals with cognitive dysfunction often show scores in the normal range [9]. In addition, some changes in ACE-R fail to accurately reflect the original ACE. For example, spelling of the word “WORLD” backwards can be substituted for subtraction of serial 7 s from 100 in ACE-R, but these two items are known to present different challenges [10]. Most importantly, ACE-R included several elements of the MMSE. Due to copyright issues, it has become difficult to keep using ACE-R. [11]

Therefore, the original authors developed a new version of ACE, namely ACE-III [6]. ACE-III is also scored on a total of 100 and contains five cognitive domains. To date, versions of ACE-III in many different languages have been validated [12–15], and we recently created a Japanese version of ACE-III.

In this study, we hypothesized that ACE-III would be superior to the conventional Hasegawa Dementia Scale-revised (HDS-R) and Mini-mental State Examination (MMSE) in detecting MCI and dementia in a Japanese population. Our objective therefore was to (1) provide detailed normative data for the sub- and total scores on ACE-III; (2) decide the optimal cut-off scores of ACE-III to identify MCI or dementia, and compare its validity with that of MMSE or HDS-R; and (3) evaluate the test-retest and inter-rater reliabilities and internal consistency.

## Methods

### Participants

A total of 389 subjects at the Memory Clinic of Okayama University Hospital between January 2013 and March 2017 who fulfilled the following criteria were included in this study (Table 1). All subjects (i) received general physical and neurological examinations and laboratory testing, including syphilis serology, plasma vitamin B1, serum vitamin B12, and thyroid function tests; (ii) took MMSE [16, 17]^,^ and HDS-R [18, 19]; and (iii) received magnetic resonance imaging (MRI) and/or computed tomography (CT) of the head. The exclusion criteria were (i) the presence of delirium or (ii) the existence of psychiatric diseases.

**Table 1:** Comparison of demographic data, MMSE, HDS-R, ACE-III total and subscores in control, MCI, and dementia groups (*n* = 389, standard deviation in parenthesis)

	CONTROL	MCI	DEMENTIA	D vs. C	D vs. M	M vs. C
	*n* = 74	*n* = 137	*n* = 178	*P* values	*P* values	*P* values
Sex, men/women	30/44	71/66	66/112	n.s.	*	n.s.
Age	72.1 (7.1)	75.3 (8.3)	78.6 (7.2)	***	***	*
Education, years	12.9 (2.3)	13.0 (2.7)	12.0 (2.4)	*	**	n.s.
MMSE, 0–30	28.5 (1.6)	25.5 (2.4)	21.7 (3.3)	***	***	***
HDS-R, 0–30	27.8 (2.2)	23.5 (3.3)	18.7 (3.8)	***	***	***
ACE-III total score, 0–100	93.5 (3.4)	82.7 (7.2)	66.0 (11.4)	***	***	***
Attention and Orientation, 0–18	17.3 (1.1)	16.0 (1.8)	13.0 (2.7)	***	***	***
Memory, 0–26	23.6 (2.2)	16.8 (4.2)	9.8 (4.3)	***	***	***
Fluency, 0–14	11.4 (1.7)	10.2 (2.3)	7.8 (2.7)	***	***	**
Language, 0–26	25.6 (0.8)	24.6 (1.7)	21.8 (3.9)	***	***	n.s.
Visuospatial, 0–16	15.6 (0.7)	15.1 (1.4)	13.6 (2.9)	***	***	n.s.

*MMSE* Mini-mental State Examination, *HDS-R* Hasegawa Dementia Scale-revised, *ACE-III* Addenbrooke’s Cognitive Examination-III, *MCI* mild cognitive impairment, *D* dementia, *C* control, *M* MCI, *n.s.* not significant

*P < 0.05, **P < 0.01, ***P < 0.001. Pairwise comparisons were performed using Bonferroni’s test

The profile of each subject (sex, age, years of education) was checked. Neuropsychological examinations were performed by clinical psychologists specialized in dementia, and the Clinical Dementia Rating (CDR) [20] score was determined by the chief clinician. When all examinations had been completed, two or more geriatric psychiatrists and two or more experienced clinical psychologists conferred, and the clinical diagnosis was established independent of the performance on ACE-III.

A total of 389 subjects were divided into three groups: a dementia group (*n* = 178), an MCI group (*n* = 137), and a control group (*n* = 74).

All patients diagnosed with dementia had a dementia severity of 0.5 (suspicious) or 1 (mild) based on the CDR. Patients in the dementia group were diagnosed with Alzheimer’s disease dementia (ADD; *n* = 131), dementia with Lewy bodies (DLB; *n* = 21), behavioral variant frontotemporal dementia (bvFTD; *n* = 9), vascular dementia (VaD; *n* = 4), and others (*n* = 13). Patients with ADD were diagnosed with probable AD according to the criteria formulated by the National Institute on Aging-Alzheimer’s Association [21]. Patients with DLB, FTD, or VaD were diagnosed in accordance with the DLB diagnostic criteria formulated by McKeith et al. [22], the FTDC criteria for bvFTD [23], and the American Heart Association/American Stroke Association guidelines for VaD [24], respectively.

Patients of the MCI group fulfilled the criteria of (1) concern about a deficit in cognition compared with the person’s previous level; (2) performance that is lower than would be expected for the patient’s age and educational background (CDR score = 0.5) in one or more cognitive domains; (3) no or minimal disturbance in activities of daily living, as established by an interview with the patient and an informant [25]; and (4) being not sufficiently functionally and cognitively impaired to meet the DSM-IV-TR criteria for dementia [26].

Seventy-four subjects with no decline in cognition compared with their previous level (CDR score = 0) were used as a control group. None had evidence of organic dementing disorders or psychiatric diseases, and all had no impairment in their activities of daily living (ADL) and instrumental ADL.

### Instruments

Like ACE-R, ACE-III is composed of five domains and each represents a certain cognitive function: (1) orientation and attention (18 points), (2) memory (26 points), (3) fluency (14 points), (4) language (26 points), and (5) visuospatial function (16 points). The highest possible score on ACE-III is 100 points, and lower scores indicate worse cognitive functioning.

The differences between ACE-R and ACE-III are as follows [2, 6]. In the attention domain of ACE-R, serial subtraction of 7 s from 100 could be replaced by spelling the word ‘WORLD’ backwards. However, in ACE-III, the option of spelling ‘WORLD’ backwards was removed, and only subtraction of serial 7 s from 100 is performed [2, 6]. In the language domain, the three-step command was changed to the three single-step commands that increase in syntactical complexity.^2^ Comprehension of the written command (‘close your eyes’) was taken out. The sentence-writing task was modified, and participants are asked to write two or more sentences on a single topic for a maximum score of 2 points [2]. The phrase repetition items were replaced by the repetition of two common proverbs [2]. Overlapping infinity loops replaced the intersecting pentagons in the visuospatial section [2]. The memory and fluency domains in ACE-R were not modified. The translation and modification of ACE-R into the Japanese version was previously reported in detail [7]. The Japanese version of ACE-III (ACE-III-J) was modified to reflect the English version of ACE-III.

MMSE is a concise cognition screening test. It includes a series of items that measure orientation, recall, language, and visual construction [16, 17]. The full score of MMSE is 30 points. HDS-R assesses cognitive function of orientation, memory, attention/calculation, delayed recall, and verbal fluency [18, 19]. This is a reliable and brief instrument to evaluate global cognitive function. The maximum total possible score is 30 points.

### Reliability

Inter-rater reliability was measured by determining the intraclass correlation coefficient (ICC) of 25 consecutive patients. Two clinical psychologists assessed subjects at the same time, and they were blind to each other’s scores. One of them actively assessed ten patients while the other passively observed, and their roles were reversed for the other 15. We evaluated test-retest reliability using the ICC of 26 consecutive patients. The second session for test-retest reliability was done four to eight weeks after the first session. We evaluated the internal consistency reliability within ACE-III-J using Cronbach’s coefficient alpha [27].

### Statistical analysis

Statistical analyses were performed using the IBM SPSS Statistics 23.0 software program. A value of *P* < 0.05 was accepted as significant. Two groups were compared by independent sample t-tests. Three groups were compared using one-way analysis of variance, followed by Bonferroni correction at the time of post hoc analysis. χ^2^ tests were used for comparison of categorical data (gender). We used a multiple regression analysis to examine possible associations of the clinical characteristics (gender, age, and years of education) with the total ACE-III score.

We determined the sensitivity and specificity of ACE-III, MMSE, and HDS-R using a receiver operating characteristic (ROC) curve [7]. We used the area under the curve (AUC) as a scale of each test’s ability to differentiate between groups of participants (dementia vs. MCI and normal; MCI vs. normal).

In this study, we used StAR software to assess statistical differences between AUCs of the three tests [28]. The most suitable cut-off scores for identifying dementia and MCI were determined as the scores that led to the maximal accuracy of classification. Subsequently, positive predictive values (PPV) and negative predictive values (NPV) were estimated at different prevalence rates (5, 10, 20, and 40%) for each optimal cutoff score.

Correlation between the CDR sum of box (CDR SoB) score and ACE-III scores was evaluated using Spearman’s correlation coefficient. A value of *P* < 0.05 was accepted as significant.

## Results

### Clinical characteristics of dementia, MCI, and control groups

Table 1 shows the clinical characteristics, MMSE scores, HDS-R scores, and ACE-III-J total and subdomain scores of dementia, MCI, and control groups.

Age (F (2, 386) = 20.93, *P* < 0.001) and years of education (F (2, 386) = 7.47, *P* = 0.001) were significantly different between the three groups. The dementia group was significantly older and less educated than the control and MCI groups, and the MCI group was older than the control group. The multiple regression analysis showed that age (β; standard partial regression coefficient = − 0.282, *P* < 0.001) and education (β = 0.129, *P* < 0.05) had a significant impact on the ACE-III-J score. When the same analysis was done on the normal controls (*n* = 74), it revealed that only age (β = − 0.266, *P* < 0.05) affected ACE-III-J performance significantly.

ACE-III-J total (F (2, 386) = 288.562, *P* < 0.001), MMSE (F (2, 386) = 184.793, *P* < 0.001), and HDS-R (F (2, 386) = 189.996, *P* < 0.001) scores were significantly different between the three groups. On ACE-III-J, scores of all five subdomains differed significantly among the three groups. According to the post hoc analysis with Bonferroni correction, the control and MCI groups had higher scores in all five domains than the dementia group (*P* < 0.001). The control group had higher scores than the MCI group in attention/orientation, memory, and fluency domains, but the differences between the two groups in language and visuospatial scores were not significant.

### Demographics of dementia group (very mild and mild)

The dementia group (*n* = 178) was subdivided into two groups, very mild (CDR = 0.5) and mild (CDR = 1), according to the CDR score. The clinical characteristics are shown in Table 2.

**Table 2:** Comparison of demographic data, MMSE, HDS-R, ACE-III total and subscores in very mild (CDR 0.5) and mild (CDR 1) dementia groups (n = 178, standard deviation in parenthesis)

	VERY MILD	MILD	*P* values
	*n* = 113	*n* = 65	
Sex, men/women	43/70	23/42	n.s.
Age	77.6 (7.7)	80.4 (6.0)	*
Education, years	11.8 (2.3)	12.3 (2.5)	n.s.
MMSE, 0–30	22.5 (3.0)	20.2 (3.1)	***
HDS-R, 0–30	19.7 (3.3)	16.9 (4.0)	***
ACE-III total score, 0–100	68.7 (10.2)	61.1 (11.8)	***
Attention and Orientation, 0–18	13.8 (2.4)	11.7 (2.6)	***
Memory, 0–26	10.1 (4.2)	9.1 (4.3)	n.s.
Fluency, 0–14	8.2 (2.5)	7.1 (3.0)	*
Language, 0–26	22.5 (3.4)	20.5 (4.4)	**
Visuospatial, 0–16	14.1 (2.4)	12.7 (3.3)	**

*MMSE* Mini-mental State Examination, *HDS-R*, Hasegawa Dementia Scale-revised, *ACE-III* Addenbrooke’s, Cognitive Examination-III; *CDR* clinical dementia rating, *n.s.* not significant

*P < 0.05, **P < 0.01, ***P < 0.001

There were no significant differences in education or gender distribution between the groups. The mild dementia group was significantly older than the very mild dementia group (*P* < 0.05) and had significantly lower scores than the very mild dementia group on ACE-III-J, MMSE, and HDS-R (*P* < 0.001). On four of the subscores of the ACE-III-J, excluding the memory score, the mild dementia group had significantly lower scores than the very mild dementia group.

### Normative data

Normative scores were generated for the ACE-III-J total and subdomain scores using data of the control group, based on the mean minus two standard deviations (lower limits of normal) for three age bands (≤69, 70–79, and ≥ 80 years old) as well as all age groups, as shown in Table 3.

**Table 3:** Lower limit of normal (cut-off scores) of ACE-III total and subscores for age bands (< 69, 70–79, > 80 years old) and all age groups (control mean - 2 standard deviations)

AGE	N	EDUCATION	TOTAL	ATTENTION	MEMORY	FLUENCY	LANGUAGE	VISUO
(range)		(mean, years)	(max. 100)	(max. 18)	(max. 26)	(max. 14)	(max. 26)	(max. 16)
- 69	26	14.0	89	16	20	10	25	16
70–79	39	12.6	87	16	20	8	24	14
80 -	9	11.6	85	15	19	8	24	14
All	74	12.9	87	16	20	8	24	15

*ACE-III* Addenbrooke’s Cognitive Examination-III, Visuo, visuospatial

Among the three age groups, the number of years of education differed (F (2, 71) = 5.228, *P* < 0.01). By post hoc analysis, the ≤69 age group had more years of education than the 70–79 and ≥ 80 age groups (respectively, *P* = 0.036 and 0.016).

Among these three age groups, ACE-III-J total scores (F (2, 71) = 3.857, *P* < 0.05) and visuospatial subscores (F (2, 71) = 4.031, *P* < 0.05) differed. No significant differences were found between the three groups in the attention/orientation (F (2, 71) = 0.279, *P* = 0.757), memory (F (2, 71) = 1.173, *P* = 0.315), fluency (F (2, 71) = 1.900 *P* = 0.157), and language (F (2, 71) = 0.174, *P* = 0.841) subscores. In the total ACE-III-J score, post hoc analysis disclosed no significant difference among the three groups. The scores of the ≤69 age group were significantly higher than those of the ≥80 age group (*P* < 0.05) in the visuospatial domain.

### Diagnostic interpretation

**Fig. 1:**
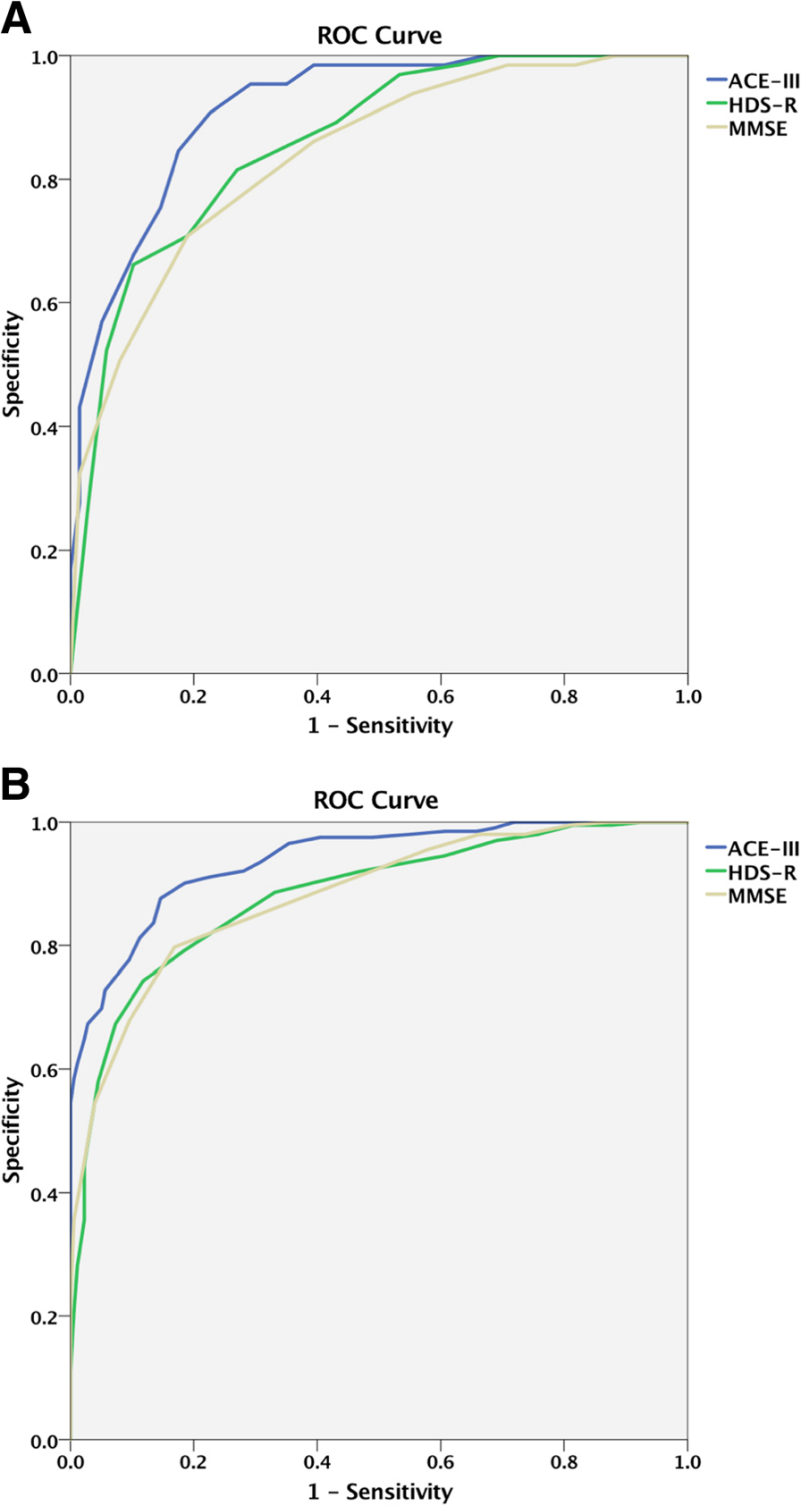
Comparative AUROCs of tests for diagnosis of each condition. A (upper). ROC curves for discriminating MCI from controls using ACE-III, HDS-R, and MMSE. B (lower). ROC curves for discriminating dementia from non-dementia (controls and MCI), using ACE-III, HDS-R, and MMSE

The ROC curves of ACE-III-J, HDS-R, and MMSE for diagnosing MCI or dementia are shown in Fig. 1 (for MCI in Fig. 1a, and for dementia in Fig. 1b). The AUCs of ACE-III-J, HDS-R, and MMSE for diagnosing MCI were 0.914 (0.876–0.953), 0.859 (0.807–0.912), and 0.838 (0.780–0.896), respectively. The AUC of ACE-III-J was significantly larger than those of HDS-R and MMSE (ACE-III-J vs. HDS-R, *P* < 0.05; ACE-III-J vs. MMSE, *P* < 0.01). The most suitable cut-off score of ACE-III-J for discriminating MCI patients from controls was 88/89 (sensitivity 0.77, specificity 0.92), and those of the HDS-R and MMSE were 24/25 (sensitivity 0.57, specificity 0.89) and 26/27 (sensitivity 0.61, specificity 0.86), respectively. The PPV and NPV of ACE-III for identifying MCI at different prevalence rates were 5% (PPV 0.35, NPV 0.99), 10% (PPV 0.52, NPV 0.97), 20% (PPV 0.72, NPV 0.94), and 40% (PPV 0.87, NPV 0.86) (Table 4).

**Table 4:** Sensitivity, specificity and positive predictive values (PPV) at different prevalence rates of optimal cut off ACE-III, HDS-R, and MMSE scores for identifying MCI and dementia (Values in parentheses represent the respective negative predictive value)

TEST	CUT-OFF	MCI		PPV at different prevalence rates			
	SCORE	SENSITIVITY	SPECIFICITY	5%	10%	20%	40%
ACE-III	88/89	0.77	0.92	0.35 (0.99)	0.52 (0.97)	0.72 (0.94)	0.87 (0.86)
HDS-R	24/25	0.57	0.89	0.22 (0.98)	0.37 (0.95)	0.57 (0.89)	0.78 (0.76)
MMSE	26/27	0.61	0.86	0.19 (0.98)	0.33 (0.95)	0.52 (0.90)	0.75 (0.77)
TEST	CUT-OFF	DEMENTIA		PPV at different prevalence rates			
	SCORE	SENSITIVITY	SPECIFICITY	5%	10%	20%	40%
ACE-III	75/76	0.82	0.90	0.30 (0.99)	0.48 (0.98)	0.67 (0.95)	0.85 (0.88)
HDS-R	20/21	0.67	0.89	0.24 (0.98)	0.39 (0.96)	0.59 (0.91)	0.80 (0.80)
MMSE	23/24	0.64	0.87	0.21 (0.98)	0.36 (0.96)	0.55 (0.91)	0.77 (0.78)

*ACE-III* Addenbrooke’s Cognitive Examination-III, *HDS-R* Hasegawa Dementia Scale-revised, *MMSE* Mini-mental State Examination

The AUCs of ACE-III-J, HDS-R, and MMSE for diagnosing dementia were 0.938 (0.915–0.960), 0.881 (0.847–0.915), and 0.881 (0.847–0.914), respectively. The AUC of ACE-III-J was significantly larger than those of HDS-R and MMSE (ACE-III-J vs. HDS-R, *P* < 0.001; ACE-III-J vs. MMSE, *P* < 0.001). For discriminating dementia patients from MCI patients and controls, the most suitable cut-off score of ACE-III-J was 75/76 (sensitivity 0.82 and specificity 0.90), and those of HDS-R and MMSE were 20/21 (sensitivity 0.67, specificity 0.89) and 23/24 (sensitivity 0.64, specificity 0.87), respectively. The PPV and NPV of ACE-III for identifying dementia at different prevalence rates were 5% (PPV 0.30, NPV 0.99), 10% (PPV 0.48, NPV 0.98), 20% (PPV 0.67, NPV 0.95), and 40% (PPV 0.85, NPV 0.88) (Table 4).

### Reliability

The inter-rater reliability of ACE-III-J was very good, with an ICC of 0.996. The test-retest reliability of ACE-III-J was also very good (ICC = 0.918). The internal consistency of ACE-III-J was high (Cronbach’s coefficient α = 0.870).

### ACE-III scores and CDR sum of boxes

Spearman’s correlation analysis of the scores of the CDR SoB and the ACE-III scores revealed that there was a significant correlation between them (correlation coefficient = − 0.396, *p* < 0.001) in MCI patients.

## Discussion

The reliability of ACE-III-J was excellent. ACE-III-J was found to be a sensitive and specific screening test to diagnose MCI and dementia in a Japanese sample, and it was better than the MMSE and HDS-R in accuracy for identifying MCI and dementia. These results suggest that ACE-III-J is a reliable and valid screening instrument.

Although ACE-III-J takes slightly longer to perform than MMSE and HDS-R, it evaluates a broader range of cognitive functions than MMSE and HDS-R, particularly in the domains of memory, language, and visuospatial components [7]. Thus, we consider that ACE-III-J provides a more useful and precise instrument than MMSE and HDS-R for diagnosing MCI and dementia. However, in 19 of the subjects, screening results for dementia are positive in MMSE but negative in ACE-III. Six of the 19 persons were diagnosed as dementia. Even if a person takes a score that exceeds the cut-off score in ACE-III, it is necessary to consider the possibility of dementia if the MMSE score of the person is below the cut-off score for dementia in MMSE.

Several non-English versions of the ACE-III have been reported [12, 14, 15, 29]. The mean scores of controls in various studies were 95.4 points (mean age 66.1 years, education 13.9 years) on the English version [6], 96.7 points (mean age 66.6 years) on the English version [30], 89.4 points (mean age 70.4 years, education 6.2 years) on the Portuguese version [12], 89.4 points (mean age 65.8 years, education 12.4 years) on the Spanish version [29], 89.3 points (mean age 68.7 years, education 11.5 years) on the Chinese version [15], and 93.5 points (mean age 72.1 years, education 12.9 years) on the Japanese version. Age and years of education had significant effects on the total ACE-III score, as shown in several studies including ours [15, 29], and those two factors might explain the differences in mean scores to some extent. The total and subscores of the control group of ACE-III-J were almost identical to those of the original English version. The mean scores of ACE-III-J vs. the original ACE-III were 93.5 vs. 95.4 in total score, 17.4 vs.17.3 in the attention domain, 25.6 vs. 25.6 in the language domain, and 15.6 vs. 15.6 in the visuospatial domain. The memory and fluency subscores in the original English version were not reported in the first paper [6].

The cut-off scores of the original ACE-III scores were almost identical to those of the original ACE-R [6]. Thus, the Japanese version of ACE-III was equivalent to the original English version as a cognitive screening instrument. The optimal cut-off score for identifying MCI (88/89) in this study was similar to the original higher cut-off score (88) for identifying dementia. The cut-off score for identifying dementia (75/76) in this study was lower than the original lower cut-off score (82). The original study for ACE-R compared dementia patients with normal controls rather than MCI patients [5]. In this study, we set an optimal cut-off score to differentiate dementia patients from MCI patients. The difference in comparative groups tested to create the cut-off scores of English and Japanese versions may have caused the difference in cut-off scores.

The sensitivities reported by other studies in which the sensitivity of the dementia diagnosis was evaluated are higher than that (0.82) in this report. In particular, Elamin et al. reported that the sensitivity for the diagnosis of dementia was 0.915, and Wang et al. reported that the sensitivity was 0.911. However, they calculated the sensitivity in distinguishing dementia patients from cognitively normal subjects or patients with subjective memory impairment in previous reports [15, 30]. In this study, in contrast, we evaluated the sensitivity in distinguishing dementia patients from MCI patients and normal subjects. The difference in the targeted patients might have caused the difference in sensitivity scores.

One study has reported the ability of ACE-III to discriminate MCI from normal controls. Matias-Guiu et al. showed that ACE-III scores discriminated between controls and amnestic MCI with high accuracy (AUC, 0.906 by ACE-III memory score) [29]. In our study, the ACE-III-J score (total score) also accurately discriminated MCI from controls (AUC, 0.914). The study of Matias-Guiu et al. detected the difference between amnestic MCI and normal controls. Therefore, the ACE-III memory score was thought to be sensitive enough to discriminate the difference. In this study, the discrimination between MCI (amnestic and non-amnestic) and normal controls was evaluated, and the total score of ACE-III-J was thought to be sensitive enough to differentiate MCI from normal controls.

Although discrimination of dementia patients from normal controls was reported by several studies [15, 29], the discrimination between dementia and MCI patients was evaluated in only one study [29]. Matias-Guiu et al. reported that ACE-III scores discriminated MCI and dementia patients with high accuracy (AUC, 0.852 by ACE-III total score). In this study, ACE-III-J total score also differentiated dementia patients from those with MCI with high accuracy (AUC, 0.938).

In the cases diagnosed with MCI in this study, the higher the CDR SoB scores were, the lower the ACE-III scores were. Kim et al. reported that the CDR SoB score is useful for predicting the progression to dementia in amnestic MCI individuals. MCI cases with a low ACE-III score may be particularly susceptible to developing dementia in the future [31].

This study has several limitations. First, there were only a few patients with dementia with Lewy bodies, vascular dementia, or frontotemporal dementia in our study. Therefore, we were unable to evaluate the differences in test scores of different dementias. Further study is needed to clarify whether or not it is possible to differentiate dementing diseases by ACE-III-J scores. Second, the participants in this study were outpatients at a university memory center. Third, we diagnosed dementia comprehensively including not only MMSE and HDS-R scores but also total living functions. However, it is undeniable that a potential circularity problem may exist. Thus, the reliability and applicability of ACE-III-J in community samples need further study.

## Conclusions

Regardless of the some limitation, ACE-III-J is an accurate instrument to detect MCI and dementia. ACE-III-J may be widely useful in clinical practice.

## Abbreviations

ACE-III: Addenbrooke’s Cognitive Examination III
ACE-III-J: The Japanese version of ACE-III
ACE-R: ACE Revised version
ADD: Alzheimer’s Disease Dementia
ADL: Activities of Daily Living
AUC: Area Under the Curve
AUROC: Area Under the Receiver Operating Characteristic curve
bvFTD: Behavioral variant Frontotemporal Dementia
CDR: Clinical Dementia Rating
DLB: Dementia with Lewy bodies
HDS-R: Hasegawa Dementia Scale-Revised
ICC: Intraclass Correlation Coefficient
MCI: Mild Cognitive Impairment
MMSE: Mini-mental State Examination
ROC: Receiver Operating Characteristic
VaD: Vascular Dementia
